# Practical Notes on Diagnosis and Treatment

**Published:** 1906-09-22

**Authors:** 


### PRACTICAL NOTES ON DIAGNOSIS AND TREATMENT.

**Eczema at the Menopause.**

FOR the eczematous and other eruptions which, together with flushings and other vaso-motor disturbances, often attend the menopause, ichthyol in doses of 5 to 10 grains is often very useful.

**Pain of Gastric Ulcer.**

THIS can often be successfully treated by sodium bicarbonate ordered in large doses. A teaspoonful of the salt is dissolved in water, and this solution is sipped at short intervals until the pain is relieved.

**Cyanosis in Phenacetin Poisoning.**

A CASE of paroxysmal cyanosis attended with hæmoglobinuria proved to be due to large doses of phenacetin which the patient took with a view to produce the above symptoms. — *Sir Lauder Brunton.*

**Urotropine.**

UROTROPINE is best prescribed either in a simple aqueous solution or in cachets. It is very soluble in water, but from the nature of its chemical constitution is a rather sensitive compound. It is not compatible with acids or acid salts. As it is precipitated by tannin, it should never be prescribed with astringent vegetable infusions or tinctures.

**Chronic Diarrhœa.**

IN very obstinate cases of chronic or recurring diarrhœa, where the stools contain much mucus, useful remedies are copper sulphate (one-third to half a grain) and castor oil. Ten minims of the oil in mucilage, or in capsule, with a minim of tincture of opium may be ordered thrice daily. Should this not succeed, one drachm of the oil should be given every morning.—*Sir Lauder Brunton.*

**Foods to Avoid in Kidney Disease.**

THERE are certain articles that should be avoided in all cases of kidney disease. One is alcohol in all forms; another is meat extracts, whether in the form of soups or broths or of the much advertised and popular extracts of meat; so also fruit and vegetables which are rich in irritating salts—*e.g.* tomatoes, asparagus, gooseberries—and, for a similar reason, salted and preserved meats.

